# Interleukin-20 Acts as a Promotor of Osteoclastogenesis and Orthodontic Tooth Movement

**DOI:** 10.1155/2021/5539962

**Published:** 2021-05-26

**Authors:** Yuanbo Liu, Yilong Ai, Xuan Sun, Bowen Meng, Xi Chen, Dongle Wu, Lei Gan, Benyi Yang, Chaoran Fu, Yilin Wu, Yang Cao

**Affiliations:** ^1^Hospital of Stomatology, Guanghua School of Stomatology, Sun Yat-sen University, Guangzhou, China; ^2^Guangdong Provincial Key Laboratory of Stomatology, Guangzhou, China; ^3^Foshan Stomatological Hospital, School of Stomatology and Medicine, Foshan University, Foshan, Guangdong, China

## Abstract

**Objectives:**

Bones constitute organs that are engaged in constant self-remodelling. Osteoblast and osteoclast homeostasis during remodelling contribute to overall skeletal status. Orthodontics is a clinical discipline that involves the investigation and implementation of moving teeth through the bone. The application of mechanical force to the teeth causes an imbalance between osteogenesis and osteogenesis in alveolar bone, leading to tooth movement. Osteoimmunology comprises the crosstalk between the immune and skeletal systems that regulate osteoclast–osteoblast homeostasis. Interleukin- (IL-) 20, an IL-10 family member, is regarded as a proinflammatory factor for autoimmune diseases and has been implicated in bone loss disease. However, the mechanism by which IL-20 regulates osteoclast differentiation and osteoclastogenesis activation remains unclear. This study investigated the effects of IL-20 on osteoclast differentiation in a rat model; it explored the underlying molecular mechanism in vitro and the specific effects on orthodontic tooth movement in vivo.

**Methods:**

For in vitro analyses, primary rat bone marrow-derived macrophages (BMMs) were prepared from Sprague–Dawley rats for osteoclast induction. After BMMs had been treated with combinations of recombinant IL-20 protein, siRNA, and plasmids, the expression levels of osteoclast-specific factors and signalling pathway proteins were detected through real-time polymerase chain reaction, western blotting, and immunofluorescence staining. For in vivo analyses, IL-20 was injected into the rat intraperitoneal cavity after the establishment of a rat orthodontic tooth movement (OTM) model. OTM distance was detected by Micro-CT and HE staining; the expression levels of protein were detected through immunofluorescence staining.

**Results:**

In vitro analyses showed that a low concentration of IL-20 promoted preosteoclast proliferation and osteoclastogenesis. However, a high concentration of IL-20 inhibited BMM proliferation and osteoclastogenesis. IL-20 knockdown decreased the expression of osteoclast specific-markers, while IL-20 overexpression increased the expression of osteoclast specific-markers. Furthermore, IL-20 regulated osteoclast differentiation through the OPG/RANKL/RANK pathway. Overexpression of IL-20 could significantly upregulate RANKL-mediated osteoclast differentiation and osteoclast specific-marker expression; moreover, RANKL/NF-*κ*B/NFATc1 acted as downstream signalling molecule for IL-20. In vivo analysis showed that OTM speed was significantly increased after intraperitoneal injection of IL-20; additionally, mechanical stress sensing proteins were markedly activated.

**Conclusions:**

IL-20 augments osteoclastogenesis and osteoclast-mediated bone erosion through the RANKL/NF-*κ*B/NFATc1 signalling pathway. IL-20 inhibition can effectively reduce osteoclast differentiation and diminish bone resorption. Furthermore, IL-20 can accelerate orthodontic tooth movement and activate mechanical stress sensing proteins.

## 1. Introduction

Osteoclasts constitute a core component of the bone multicellular unit. They have a vital role in bone remodelling and are an essential role in the maintenance of skeletal structural integrity and metabolic capacity [1–3]. The coordinated functions of skeletal cells are regulated by multiple hormones, growth factors, chemokines, and cytokines that act via interconnected signalling networks, resulting in the activation of specific transcription factors and their corresponding target genes [4]. Receptor activator of NF-кB ligand (RANKL) and macrophage colony-stimulating factor (M-CSF) are secreted and expressed by various cells including osteoblasts; these are key factors in osteoclastogenesis and bone resorption [5, 6].

In the context of osteoimmunology, cytokine crosstalk during osteoclast differentiation is receiving increasing attention. Many studies have indicated that immune cells, such as macrophages and T cells, secrete proinflammatory cytokines (e.g., IL-1, IL-6, IL-10, IL-17, IL-18, IL-22, IL-33, and TNF), which are involved in mediating osteoclastogenesis [7–13]. IL-20 is considered a proinflammatory factor in the context of autoimmune diseases; it also acts as an IL-10-related immunoregulatory molecule [14]. Notably, IL-20 can induce synovial fibroblasts to produce proinflammatory molecules, including TNF-*α*, IL-1*β*, MMP-1, MMP-13, and MCP-1. This effect activates T cells, monocytes, dendritic cells, and neutrophils, thus, causing tissue and bone damage [15, 16]. Studies have shown that the median serum levels of IL-20 in patients with osteoporosis and osteopenia are 209.5 pg/mL and 181.3 pg/mL, respectively; however, healthy people exhibited a median serum level of 15.38 pg/mL. Furthermore, the use of a mouse anti-human IL-20 monoclonal antibody protected ovariectomised (OVX) mice against bone destruction, while facilitating increased bone mineral density. This antibody also inhibited IL-20-induced RANKL expression in osteoblasts [17]. These data indicate that IL-20 can inhibit osteoblasts and promote osteoclast formation. IL-20 is a decisive factor in the balance between osteoclast and osteoblast differentiation. Bone homeostasis is achieved through combined osteoclast and osteoblast activity. Additionally, we previously found that IL-20 has an inhibitory effect on the osteoblast maturation of mouse preosteogenic MC3T3-E1 cells [18]. These findings confirmed the influence of IL-20 on osteoclast differentiation and function.

Orthodontic tooth movement depends on the coordinated absorption and formation of surrounding bone and periodontal ligament tissue. Tooth loading causes local hypoxia and fluid flow, which triggers a sterile inflammatory cascade that ultimately leads to osteoclast resorption in the compression side and osteoblast deposition in the tension side. During orthodontic tooth movement, the imbalance between osteoblastogenesis and osteoclastogenesis is the basis for alveolar bone reconstruction and tooth movement [19–21]. We hypothesise that IL-20 can accelerate the speed of orthodontic tooth movement.

In this study, we investigated the effects of IL-20 on osteoclast differentiation and function through the RANKL/NF-*κ*B/NFATc1 signalling pathway. Notably, IL-20RB knockdown led to partial inhibition of the ability of IL-20 to promote osteoclast differentiation. Although the expression of NF-*κ*B did not significantly change, the expression levels of TRAF6 and NFATc1 were significantly reduced. These findings showed that IL-20 affects osteoclast differentiation by regulating the TRAF6/NFATc1 signalling pathway. Furthermore, in vivo analyses demonstrated that IL-20 can significantly enhance the orthodontic movement of teeth, and the expression levels of IL-20, TRAP, and YAP in the periodontal ligament were significantly increased in teeth undergoing orthodontic movement.

This study proved the effect of IL-20 on the differentiation and function of osteoclasts and its mechanism; it demonstrates that IL-20RB is a key factor in the effects of IL-20 on osteoclast differentiation, while providing important information for the experimental analysis of orthodontic tooth movement.

## 2. Materials and Methods

### 2.1. Cytokines, Reagents, and Antibodies

Recombinant rat M-CSF and RANKL were obtained from PeproTech (USA) and R&D Systems (USA), respectively. Recombinant rat IL-20 was purchased from Sino Biological Inc. (China). Dulbecco's modified Eagle's medium (DMEM; high glucose formulation), fetal bovine serum (FBS), phosphate-buffered saline (PBS), penicillin, and streptomycin were purchased from Invitrogen (USA). Red blood cell (RBC) lysis buffer was purchased from CWBIO (China). Primary antibodies against GRB2, ERK, NF-*κ*B, TRAP, CTSK, JNK, TRAF6, I*κ*K, and p-38 were obtained from Cell Signalling Technology Inc. (USA). Primary antibodies against RANKL, OPG, MMP-9, NFATc1, IL-20, IL-20RB, HIF-*α*, CXCR-4, CCR7, and VEGF-R2 were obtained from Abcam (USA).

### 2.2. Animals and Animal Ethics

Four-week-old Sprague–Dawley rats (*n* = 120) were obtained from the Animal Experimental Center of Sun Yat-sen University and used in this study. Rats were fed, anaesthetised, and killed in accordance with the guidelines of the Institutional Animal Care and Use Committee (IACUC) of Sun Yat-sen University. All experimental protocols were approved by the Animal Ethical and Welfare Committee of Sun Yat-sen University (SYSU-IACUC-2018-000099, Guangzhou, China).

### 2.3. Cell Isolation and Culture

Primary rat bone marrow-derived macrophages (BMMs) were obtained from the femurs and tibias of 4-week-old Sprague–Dawley rats. The methods for BMM collection were performed as described previously [22], with minor modifications. Briefly, the femur and tibia of each rat were separated; then, bone marrow cells were extracted. The cells were centrifuged at 450 g for 5 minutes and then resuspended in RBC lysis buffer on ice for 15 minutes to enable purification of bone marrow cells. Resuspended cells were centrifuged at 500 g for 10 minutes to collect BMMs and remove RBCs. Resuspended cells with primary culture medium composed of 10% FBS and DMEM (high glucose formulation) and then cultured in a humidified environment of 5% carbon dioxide and 37°C. After 48 hours of culture, nonadherent cells were collected and resuspended in an antibiotic-free complete medium containing 10% FBS and 15 ng/mL M-CSF; cells were plated in 24-well plates (2 × 10^6^ cells/well) for 2–3 days to induce BMMs to differentiate into preosteoclasts. To induce osteoclastogenesis, the osteoclast culture medium was replaced with an antibiotic-free complete medium containing M-CSF (30 ng/mL) and RANKL (50 ng/mL) at 2-day intervals for 6-8 days until the osteoclasts differentiated and matured.

### 2.4. Cell Viability Assay

Cell Counting Kit-8 reagents were purchased from Dojindo. Briefly, preosteoclasts were cultured in 96-well plates with antibiotic-free complete medium supplemented with varying concentrations of IL-20 (0.02–100 ng/mL). The absorbance in each well was measured at 450 nm with a microplate reader (Tecan SUNRISE microplate reader, Tecan, Switzerland) after 1, 3, 5, and 7 days of culture.

### 2.5. TRAP-Positive Staining and Bone Resorption Pit Assay

After culture with M-CSF and RANKL, mature osteoclasts were treated in 4% paraformaldehyde and stained with an Acid Phosphatase Leukocyte (TRAP) Kit (Sigma-Aldrich, USA), in accordance with the manufacturer's protocol. Using an inverted microscope (Zeiss, Germany), the numbers of TRAP-positive multinucleated cells with ≥3 nuclei were then counted to determine the number of osteoclast-like cells. To observe the bone resorption activity of mature osteoclasts, BMMs were cultured with M-CSF and RANKL in a 24-well osteo assay surface multiple-well plate (Corning Life Sciences, USA) coated with a thin inorganic three-dimensional crystalline material. After 6-8 days of culture, a pit formation assay was performed, and 100 *μ*L of 10% bleach solution was added to each well. Cells were then incubated in the bleach solution for 10 minutes at room temperature. The wells were rinsed twice with distilled water and allowed to air dry at room temperature for 2 hours. Using an inverted microscope (Zeiss), analyses of the individual pits or multiple pit clusters were performed at 5× magnification.

### 2.6. qRT-PCR Analysis

Primer pairs were purchased from Takara and RiboBio, and the primer sequences used for this experiment are shown in Table 1. Total mRNA was extracted with an RNA Rapid Purification Kit (ES Science, China), in accordance with the manufacturer's protocol. Assessments of extracted RNA concentration and quality were performed using NanoDrop ND-1000 Spectrophotometer analysis (NanoDrop Technologies, USA). PrimeScript™ RT Master Mix (Perfect Real Time) (Takara, Japan) was used for reverse transcription to generate cDNA. Quantitative reverse transcription-polymerase chain reaction (qRT-PCR) was performed with SYBR® Premix Ex Taq™ (Tli RNaseH Plus) (Takara, Japan) using a MicroAmp Optical 394-Well Reaction Plate with Barcodes (Thermo Fisher Scientific) on a QuantStudio 7 Flex Real-Time PCR System (Applied Biosystems™). Relative mRNA expression of target genes was calculated using the 2^−*ΔΔ*Ct^ method and normalised against the expression of the *GAPDH* housekeeping gene.

### 2.7. Western Blotting Analysis

Cell lysates were prepared using ice-cold RIPA lysis buffer in the presence of a protease/phosphatase inhibitor cocktail (Cell Signalling Technology), and the supernatants were collected for further experiments. Briefly, equal amounts (50 *μ*g) of protein samples were resolved using sodium dodecyl sulfate-polyacrylamide through 8% gel electrophoresis and then transferred to an Immobilon®-P Transfer Membrane (Millipore, USA). The membrane was blocked with 5% (*w*/*v*) bovine serum albumin (BSA) at 4°C overnight and then incubated with primary antibodies and secondary antibodies. Immunoreactivity was visualised with the Immobilon™ Western Chemiluminescent HRP substrate (Millipore).

### 2.8. Small Interfering RNA Transfection

Bone marrow-derived macrophages were transfected with Cy3-conjugated small interfering RNA (siRNA) targeting IL-20 and IL-20RB, or comprising negative control siRNA. The IL-20- and IL-20RB-targeting siRNAs and negative control siRNA were purchased from RiboBio (Guangzhou, China). The siRNA sequences are shown in Tables 2 and 3. The siRNA transfection mixture was mixed with Lipofectamine 3000 transfection reagent (Thermo Fisher Scientific, USA) in serum-free DMEM. Rat BMMs were inoculated into six-well plates (2 × 10^6^ cells/well) with antibiotic-free complete medium. After 4–6 hours, the cells were adherent; the complete medium was removed and cells were washed twice with PBS and then incubated with the transfection mixture for 6–8 hours. After incubation, the transfected cells were cultured with DMEM containing 10% FBS for 48 hours. qRT-PCR and western blotting analysis were performed as described above.

### 2.9. Plasmid Extraction and Overexpression of IL-20

Design and synthesis of overexpression gene sequences were performed by RiboBio. Sequence details are provided in Table 4. An overexpression plasmid was extracted from *Escherichia coli* DH5*α* using an EndoFree Maxi Plasmid Kit (Tiangen, China), and its concentration and quality were determined using NanoDrop ND-1000 Spectrophotometer analysis (Nanodrop Technologies). The plasmid transfection mixture was mixed with Lipofectamine 3000 transfection reagent (Thermo Fisher Scientific) in serum-free DMEM. BMM culture and analysis were performed as described above. The sequencing blow is cloned fragment sequencing results. The underlined part of the sequence is the cloned target sequence, and the upstream and downstream regions are the sequences of the vector frame.

CTATATAAGCAGAGCTCTCTGGCTAACTAGAGAACCCACTGCTTACTGGCTTATCGAAAGCTGTAATACGACTCACTATAGGGATCCCAGGAATTCGCCGCCACCATGAGAGGCTTTCGTCTTGCCTTTGGACTGTTCTCCGTTGTGGGTTTTCTTCTCTGGACTCCTTTAACTGGGCTCAAGACCCTTCATTTGGGAAGCTGTGTAATCACTGCAAACCTACAGGCGATACAAAAGGAATTTTCTGAGATTCGGCATAGTGTGCAAGCTGAAGATGAAAATATCGACGTCAGGATTTTAAGGACGACTGAGTCCCTGAAAGACACAAAGCTTTCGGATAGGTGCTGCTTTCTCCGCCATCTAGTGAGGTTCTATCTGGACAGGGTGTTCAAAGTCTACCAGACCCCTGACCATCATACCCTGCGAAAGATCAGCAGCCTCGCCAATTCTTTTCTTATCATCAAGAAGGACCTCTCAGTCTGTCATTCTCACATGGCATGTCATTGTGGCGAAGAAGCAATGGAGAAATACAACCAAATTCTCAGTCATTTCACAGAGCTTGAGCTCCAGGCAGCCGTGGTGAAGGCTTTGGGGGAACTAGGCATTCTTCTGAGATGGATGGACTCGAGTCTAGAGGGCCCGTTTAAACCCGCTGATCAGCCTCGACTGTGCCTTCTAGTGGCC.

### 2.10. Gene Ontology (GO) Analysis and Pathway Analysis

mRNA analysis included GO analysis (http://www.geneontology.org), which provides three structured networks of defined terms that describe gene product attributes. *P* values denote the significance of GO term enrichment in the predicted mRNA list, where *P* < 0.05 was considered statistically significant. The most enriched GO terms ranked by fold enrichment and enrichment score among the three groups were identified. Pathway analysis was also performed using the most current Kyoto Encyclopedia of Genes and Genomes (KEGG) database. This functional analysis allowed the identification of biological pathways for which there was a significant enrichment of differentially expressed mRNAs. As noted above, *P* < 0.05 was considered statistically significant.

### 2.11. Establishment of Rat Orthodontic Tooth Movement Model

Rats were fixed in the supine position after routine anaesthesia. We used a custom-designed force applying device, comprising a tension spring cut into small sections of approximately 5 mm, with two 0.1 mm diameter ligature wires tied to the ends of the tension springs, while the ends were permitted to remain long for ligation and fixation to the teeth. The left maxillary first molar and left maxillary incisor were cleaned and dried. One end of the force device was fixed to the maxillary first molar, and one end of the ligature was passed through the gap between the maxillary first and second molars; it was then tightened and cut. The maxillary first molar was then cleaned with alcohol, dried, and treated with an acid etching agent for 40 seconds. After full removal of the etching agent, the resin was bonded to the proximal surface of the first molar and then shaped with an oral applicator as follows. The connection between the ligation wire and tension spring was wrapped to prevent the device from loosening; the end of the ligature wire was also wrapped to prevent the end from damaging the rat's mouth. Concurrently, a small amount of resin was bonded to the occlusal surface of the molar to strengthen the retention. An orthodontic dynamometer was used to measure and record the position of the ligature wire at a tension of 50 g. The other end of the force device was then ligated and fixed to the maxillary central incisor, and the end was cut to prevent detachment of the device. Then, we used Micro-CT and HE staining to assess the rat orthodontic tooth movement model.

### 2.12. Statistical Analysis

All results are expressed as the mean ± standard deviation, and reported values were obtained from at least three experiments. Statistical differences were evaluated with GraphPad Prism software, version 7.04, using Student's *t*-test or one-way ANOVA with Tukey's post hoc analysis. *P* < 0.05 was considered statistically significant.

## 3. Results

### 3.1. BMM Proliferation Is Influenced by the Concentration of IL-20

**Figure 1:**
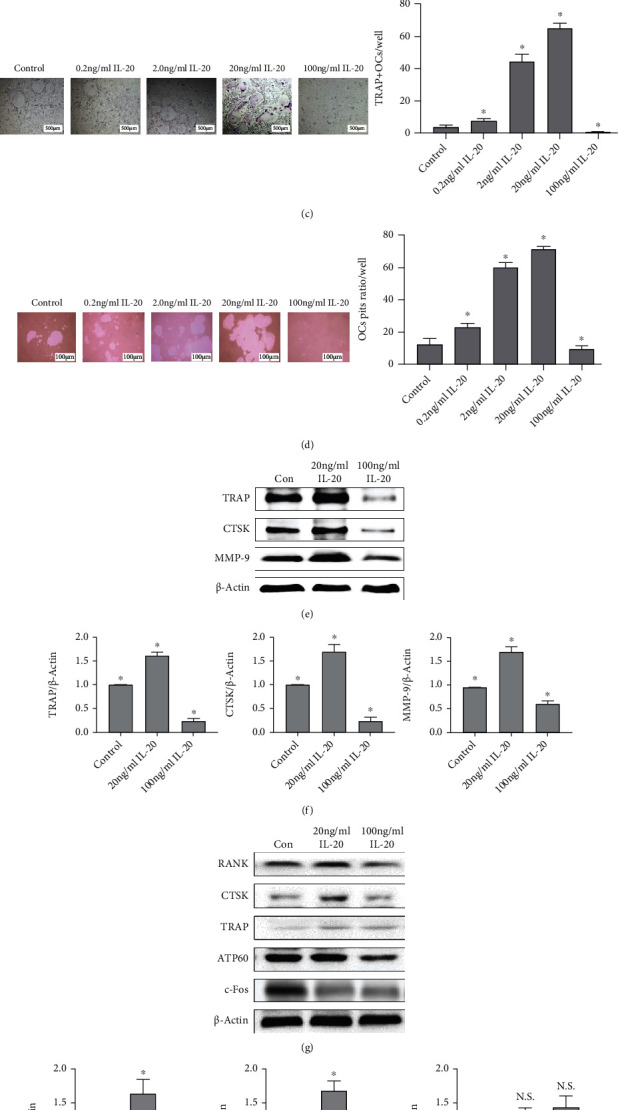
IL-20 concentration differentially regulates BMM activity, and a low concentration of IL-20 can activate ERK signalling to promote cell proliferation. BMM viability was detected by CCK8 assay. IL-20 concentration differentially regulates osteoclast formation and bone resorption capacity. (a) Cell viability analysis of IL-20-treated BMMs on days 1, 3, 5, and 7. Bars represent the mean ± SEM of six independent experiments (*n* = 12). (b) BMMs treated with various concentrations of IL-20 were used to investigate the protein expression levels of ERK, GRB2, and NF-*κ*B by western blotting during early osteoclast differentiation. Bars represent the mean ± SEM of three independent experiments (*n* = 12). ^∗^*P* < 0.05 vs. control group; ns: not significant. (c) M-CSF-induced preosteoclasts were cultured with 30 ng/mL RANKL and various concentrations of IL-20. The control group comprised M-CSF-induced preosteoclasts cultured with 30 ng/mL M-CSF, 30 ng/mL RANKL, and 0 ng/mL IL-20; TRAP-positive staining was used to examine and measure the number of TRAP-positive osteoclasts with ≥3 nuclei. (d) A bone resorption pit assay was performed to examine osteoclast function. M-CSF-induced preosteoclasts in the control group were treated as described for the TRAP staining assay; the areas of bone resorption pits were quantified. (e and f) Expression levels of osteoclast-specific proteins (TRAP, CTSK, and MMP-9) were examined using western blotting. (g and h) Expression levels of osteoclast marker genes (*RANK*, *CTSK*, *TRAP*, *ATP60*, and *c-Fos*) during early osteoclast differentiation after supplementation of IL-20 at 20 ng/mL or 100 ng/mL. Bars represent the mean ± SEM of six independent experiments (*n* = 18). ^∗^*P* < 0.05 vs. control group; N.S.: not significant.

To determine whether IL-20 can affect BMM proliferation, we treated BMMs with various concentrations of IL-20 and used a CCK-8 assay to detect cell proliferation activity on days 1, 3, 5, and 7. We found that an IL-20 concentration of 20 ng/mL was sufficient to promote BMM proliferation (Figure 1(a)). However, an IL-20 concentration of >100 ng/mL caused inhibition of BMM proliferation. Western blotting analysis confirmed that, at an IL-20 concentration of 20 ng/mL, BMM proliferation signalling factors (e.g., *GRB2*, *ERK*, and *NF-κB*) were significantly upregulated during early osteoclast differentiation (Figure 1(b)). Using the same cell treatment method, we administered various concentrations of IL-20 to M-CSF-induced preosteoclasts with RANKL and then identified the osteoclast number and size by TRAP staining after 6-8 days of cell culture. We found that an IL-20 concentration of 20 ng/mL led to significant increases in the number and size of TRAP-positive osteoclasts, compared with the control group; conversely, an IL-20 concentration of 100 ng/mL significantly reduced the number of TRAP-positive osteoclasts (Figure 1(c)). In addition, we incubated M-CSF-induced preosteoclasts with RANKL in osteo assay surface plates treated with various concentrations of IL-20. Using a bone resorption pit assay, we found that an IL-20 concentration of 20 ng/mL significantly enhanced the size of the bone resorption pit, compared with the pit sizes in other groups; however, an IL-20 concentration of 100 ng/mL led to minimal changes in the bone resorption pit area (Figure 1(d)). These results indicated that IL-20 regulated osteoclastogenesis and function in a dose-dependent manner.

Subsequently, we treated M-CSF-induced preosteoclasts with RANKL and IL-20 at a concentration of 20 ng/mL for 6–8 days. Western blotting analyses indicated that IL-20 modulated the expression patterns of osteoclast-specific and bone resorption functional proteins (e.g., TRAP, CTSK, and MMP-9) (Figures 1(e) and 1(f)). To confirm the effect of IL-20 on early osteoclast differentiation, we treated M-CSF-induced preosteoclasts with RANKL and IL-20 for 2–3 days. Western blotting analyses revealed the expression of marker genes (e.g., *RANK*, *CTSK*, *TRAP*, *ATP60*, and *c-Fos*) in early osteoclast differentiation (Figures 1(g) and 1(h)). These results indicated that, during early osteoclast differentiation, a low concentration of IL-20 upregulated the expression of *RANK* and *CTSK*, whereas it downregulated the expression of *c-Fos*; moreover, a high concentration of IL-20 downregulated the expression of *RANK*, *CTSK*, and *ATP60*. Notably, IL-20 had no effect on *TRAP* expression. These results indicated that a low concentration of IL-20 promotes early osteoclast differentiation. Furthermore, TRAP is a protein specifically expressed in mature osteoclasts; our results indicate that it exhibits minimal or no expression during early osteoclast differentiation.

### 3.2. IL-20 Modulated the Expression of Osteoclast-Specific Proteins and Promoted Osteoclastogenesis through the OPG/RANKL/RANK Axis

**Figure 2:**
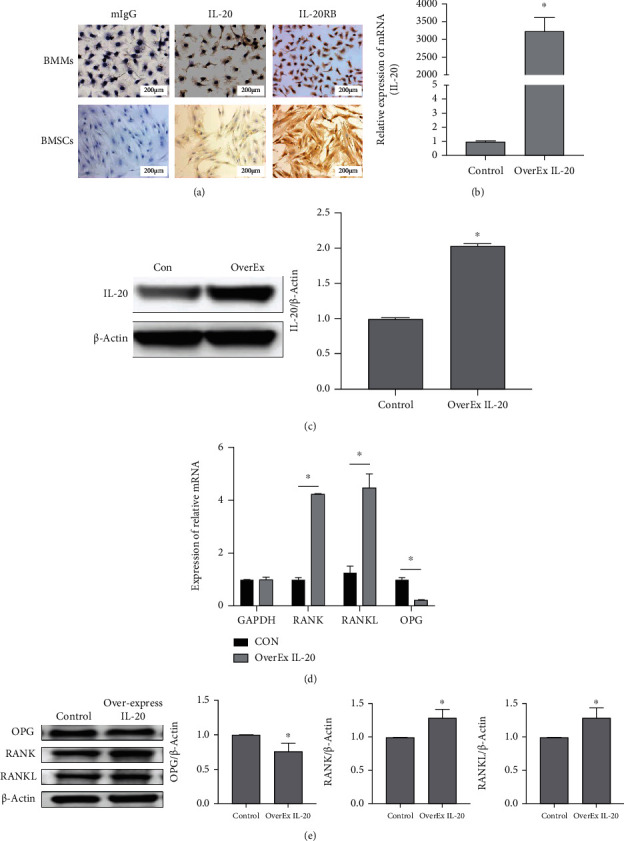
Overexpression of IL-20 regulates the expression of RANKL and OPG, indicating that IL-20 can modulate osteoclast differentiation through the OPG/RANKL/RANK axis. (a) Expression levels of IL-20 and its receptor IL-20RB in BMSCs and BMMs were determined by immunohistochemical staining. The use of an overexpression plasmid to enhance IL-20 expression in BMMs led to increased expression levels of RANKL and RANK, whereas it led to a decreased expression level of OPG. (b and c) BMMs were transfected with overexpression plasmids to increase the expression of IL-20, and the expression level of IL-20 was detected by qRT-PCR and western blotting; the control group received no plasmid. ^∗^*P* < 0.05 vs. control group. (d) mRNA expression levels of RANK, RANKL, and OPG were evaluated by qRT-PCR after 2 days of transfection. ^∗^*P* < 0.05 vs. control group; (e) protein expression levels of OPG, RANK, and RANKL were examined by western blotting. Bars represent the mean ± SEM of three independent experiments (*n* = 12). ^∗^*P* < 0.05 vs. control group; N.S.: not significant.

Using TRAP-positive staining and bone resorption pit assays, we found that IL-20 influenced osteoclastogenesis and bone resorption ability. Cellular immunohistochemistry analysis confirmed the presence of IL-20 and its receptor IL-20RB in bone marrow stromal cells (BMSCs) and bone marrow monocytes (Figure 2(a)). These results provided an experimental basis for using siRNA to knock down IL-20 and its receptor IL-20RB; it also provided a basis for performing IL-20 overexpression assays with liposomes. The OPG/RANKL/RANK axis has various cell regulatory functions, but its most well-known point is the osteoclast differentiation upstream signalling pathway. This biological axis regulates osteoclast differentiation through the antagonistic action of OPG and RANKL [8, 10, 23–27]. To investigate whether IL-20 can regulate osteoclast differentiation through the OPG/RANKL/RANK axis, we constructed an IL-20 overexpression plasmid using *E. coli* DH5*α* and then transfected the plasmid into BMMs. The expression of IL-20 in BMMs after transfection was detected by qRT-PCR and western blotting (Figures 2(b) and 2(c)). After transfection, the cells were stimulated to differentiate into osteoclasts, and the expression patterns of OPG, RANK, and RANKL were investigated using qRT-PCR and western blotting (Figures 2(d) and 2(e)). Compared with the control group, the expression levels of RANK and RANKL were significantly increased in the IL-20 overexpression group, while the expression level of OPG was significantly reduced; moreover, the RANKL/OPG ratio was significantly increased. These results clearly showed that increased expression of IL-20 could regulate the expression of RANKL and OPG, indicating that IL-20 can modulate osteoclast differentiation through the OPG/RANKL/RANK axis.

### 3.3. GO Analysis and Pathway Analysis following the Overexpression of IL-20 in Bone Marrow-Derived Mononuclear Cells

**Figure 3:**
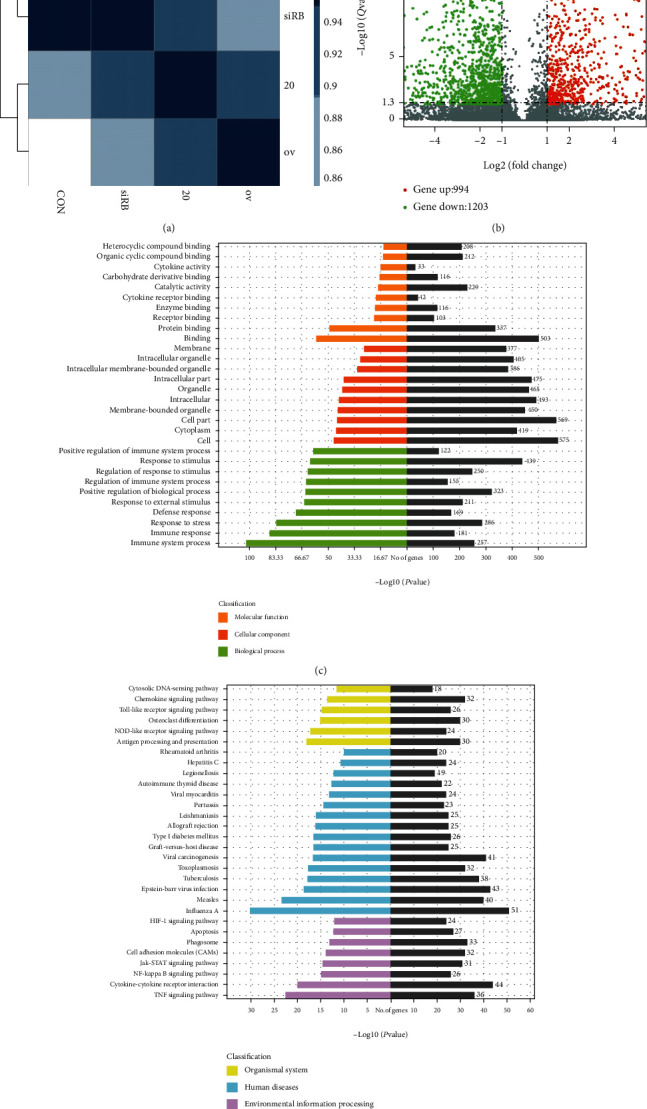
GO analysis and KEGG analysis of the difference between the mRNA of BMMs overexpressing IL-20 and normal BMMs. (a) Significant differences between cells in the control and IL-20 overexpression groups. (b) Volcano charts of differential mRNA expression. Red and green denote high and low expression levels, respectively. Each mRNA transcript is represented by a single row of coloured boxes, and each sample is represented by a single column. (c) Most significantly enriched GO (−log10 (*P* value)) terms of mRNA gene symbols according to biological process, cellular component, and molecular function. (d) Bar plot shows the top ten enrichment scores (−log10 (*P* value)) of significantly enriched pathways.

The above findings indicated that IL-20 can modulate the OPG/RANKL/RANK pathway to promote osteoclast differentiation. To further investigate the effects of IL-20 on monocytes and osteoclasts, we used high-throughput transcriptome sequencing (RNA-seq) to detect differences in mRNA expression between normal BMMs and IL-20-overexpressing BMMs. The results showed a large difference between groups, indicating that IL-20 overexpression had a significant effect on preosteoclasts (Figure 3(a)). Volcano diagram depiction revealed that, compared with normal BMMs, IL-20-overexpressing BMMs exhibited 994 significantly upregulated genes and 1203 significantly downregulated genes (Figure 3(b)). GO analysis and pathway analysis were performed to evaluate the roles of IL-20 in biological processes, cellular components, molecular functions, and pathways. GO analysis demonstrated that IL-20 overexpression had a strong effect on the monocyte biological process, and the impact was concentrated mainly on the cellular immune response and the cellular response to stimuli. Notably, IL-20 overexpression in BMMs substantially changed their response to stress, which implies that IL-20 is important in both distraction osteogenesis and orthodontic alveolar bone reconstruction (Figure 3(c)). KEGG analysis demonstrated that IL-20 overexpression in BMMs had substantial effects on osteoclast differentiation and chemokine interactions. It also had robust effects on arthritis pathogenesis, the downstream osteoclast differentiation pathway induced by RANKL, and the HIF-*α* signalling pathway and apoptosis (Figure 3(d)).

### 3.4. IL-20 Regulated RANKL-Mediated Osteoclastogenic Downstream Signal Transduction

**Figure 4:**
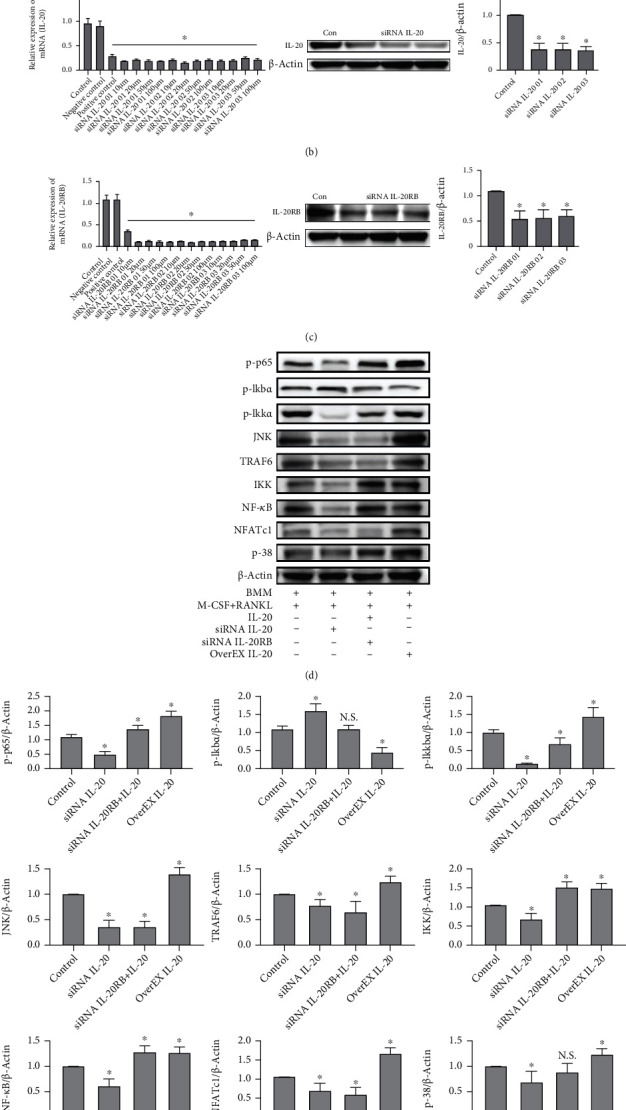
Robust expression of IL-20 significantly activated the RANKL-mediated downstream signalling pathway. siRNA reduction of IL-20 expression in BMMs led to significant inhibition of the RANKL-mediated downstream signalling pathway; moreover, siRNA reduction of IL-20RB, a key receptor for IL-20, in BMMs led to inhibition of IL-20-modulated downstream signalling pathways. (a) Bone marrow-derived mononuclear cells were transfected with siRNA carrying Cy3 dye and imaged with a fluorescence-inverted microscope to confirm the presence of siRNA within the cells. (b and c) qRT-PCR and western blotting analyses verified the inhibition of IL-20 and IL-20RB expression by siRNA at different concentrations (10–100 *μ*M of each siRNA sequence); three siRNA sequences were designed and synthesised for each gene. (d and e) Western blotting analysis was used to detect the RANKL-mediated downstream signalling pathway protein expression levels in BMMs transfected with IL-20 siRNA, IL-20RB siRNA with 20 ng/mL IL-20, and the IL-20 overexpression plasmid for 2 days; examined proteins included p-I*κ*k*α*, p-I*κ*k*β*, p-p65, JNK, TRAF6, I*κ*K, NF-*κ*B, NFATc1, and p-38. Bars represent the mean ± SEM of three independent experiments (*n* = 12). ^∗^*P* < 0.05 vs. control group; N.S.: not significant.

To explore the mechanism by which IL-20 regulates RANKL-mediated osteoclast differentiation, bone marrow-derived mononuclear cells were transfected with siRNA fluorescence staining showed high transfection efficiency (Figure 4(a)). qRT-PCR and western blotting analyses showed that siRNA had significant transfection efficiency with respect to target genes (Figures 4(b) and 4(c)). After transfection, BMMs were cultured in antibiotic-free complete medium with M-CSF and RANKL for 3 days. Western blotting revealed that, compared with the control group, cells in the IL-20 overexpression group exhibited activation of the RANK/RANKL downstream signalling effectors in osteoclastogenesis (e.g., JNK, NF-*κ*B, TRAF6, I*κ*K, NFATc1, and p38) (Figure 4(d)). In contrast, the group that received siRNA to suppress IL-20 expression showed significant inhibition of the above signalling pathways; in particular, p-I*κ*k*α* was significantly activated, indicating inhibition of the NF-*κ*B signalling pathway. In addition, we used siRNA to reduce the expression of IL-20RB and cultured the cells with an antibiotic-free complete medium containing 20 ng/mL IL-20 (Figure 4(d)). Western blotting demonstrated that the I*κ*K and NF-*κ*B signalling pathways were activated, whereas the JNK, TRAF6, NFATc1, and p-38 pathways were not (Figure 4(e)). Similar to inhibition of IL-20, the inhibition of IL-20RB also inhibited the activation of TRAF6/NFATc1 signalling pathways; this indicated that the key IL-20 receptor, IL-20RB, can regulate activation of the osteoclast differentiation signalling pathway.

### 3.5. IL-20 Feedback Regulates BMSC Involvement in Osteoclastogenesis through the OPG/RANKL/RANK Axis and Downstream Signal Transduction

**Figure 5:**
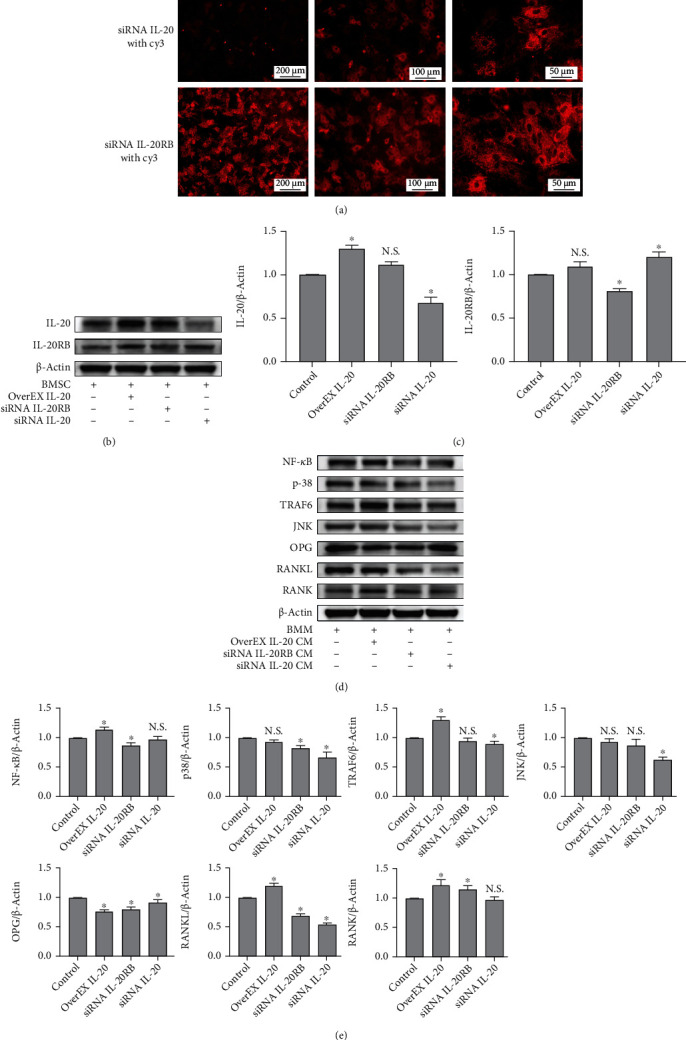
IL-20 can induce BMSCs to regulate the expression of OPG and RANKL and then influence osteoclastogenesis through the OPG/RANKL/RANK axis. (a) BMSCs were transfected with siRNA carrying Cy3 dye and imaged with a fluorescence-inverted microscope to confirm that siRNA had been transfected into the cells. (b and c) Western blotting analysis verified the inhibition of IL-20 and IL-20RB expression by siRNA and the overexpression of IL-20 using a plasmid. (d and e) Western blotting analysis detected protein levels of OPG/RANK/RANKL axis components and a subset of RANKL-mediated downstream signalling pathway components in BMMs cultured with BMSC CM treated with IL-20 siRNA, IL-20RB siRNA, and an IL-20 overexpression plasmid. Bars represent the mean ± SEM of three independent experiments (*n* = 12). ^∗^*P* < 0.05 vs. control group; N.S.: not significant.

BMSCs are cells with self-renewal ability, capable of producing at least one type of highly differentiated progeny cell with multidirectional differentiation potential; BMSCs can also produce cytokines involved in immune responses. After BMSCs differentiate into osteoblasts, M-CSF and RANKL can be secreted, and these factors can induce the formation of osteoclasts [5]. In this experiment, we used siRNA to knock down IL-20 and its key receptor IL-20RB in BMSCs; we also used a plasmid to induce BMSCs to overexpress IL-20. After transfection, BMSCs were continuously cultured for 48 hours; we then collected and used BMSC conditioned medium- (CM-) cultured BMMs. Fluorescence staining showed high transfection efficiency (Figure 5(a)). Western blotting analyses showed that siRNA had significant transfection efficiency with respect to target genes (Figures 5(b) and 5(c)). Western blotting analysis of bone marrow-derived mononuclear cells after 3 days of culture with BMSC CM showed that, compared with the control group, the expression levels of RANK and RANKL were significantly increased in BMMs cultured with IL-20-overexpressing BMSC CM; moreover, the expression of OPG was significantly reduced and the RANKL/OPG ratio was significantly enhanced (Figures 5(d) and 5(e)). BMMs cultured with BMSC CM treated with IL-20 siRNA showed significantly elevated OPG expression levels. In addition, BMMs cultured with IL-20-overexpressing BMSC CM demonstrated activation of downstream osteoclastogenesis signalling pathways mediated by the RANK/RANKL axis (e.g., NF-*κ*B and TRAF6 pathways); however, the p38 and JNK pathways were not activated. BMMs cultured with IL-20 siRNA-treated BMSC CM exhibited downregulation of the p38, TRAF6, and JNK pathways. These results indicated that IL-20 could directly induce preosteoclast differentiation into osteoclasts. Moreover, it regulated the expression of OPG and RANKL by induction of BMSCs and activation of some downstream signalling pathways that are activated by the OPG/RANK/RANKL axis in osteoclastogenesis [28], thereby indirectly promoting preosteoclast differentiation into osteoclasts.

### 3.6. IL-20 Can Accelerate the Speed of Rat Orthodontic Tooth Movement

**Figure 6:**
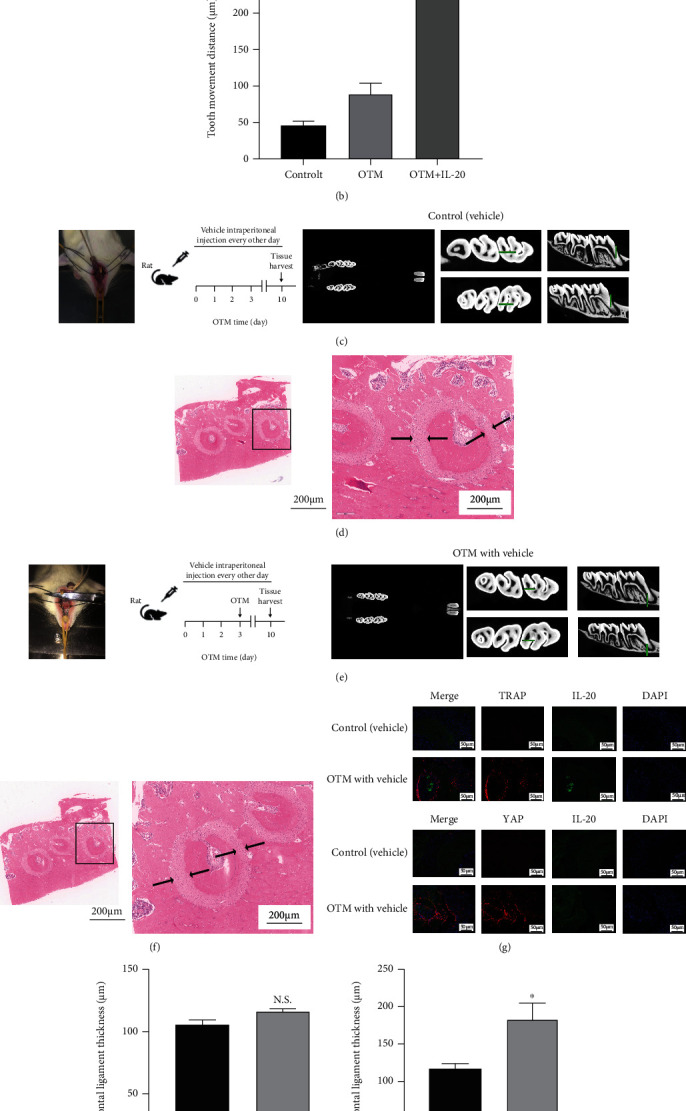
(a) OTM + IL-20 group: during the application of orthodontic force, an IL-20 solution with a concentration of 40 mg/kg body weight was injected at 2-day intervals. Seven days after the application of orthodontic force, micro-CT showed larger gaps between first and second molars. (b) Micro-CT measurement revealed that the gap between the first and second molars was significantly greater in the OTM + IL-20 group than in the OTM group. Bars represent the mean ± SEM of three independent experiments (*n* = 12). ^∗^*P* < 0.05 vs. OTM group; ns: not significant. (c) Control group: no orthodontic force was applied, and 0.9% saline alone was injected at 2-day intervals. Micro-CT showed no obvious gap between first and second molars. (d and f) HE staining showed significant changes in first molar periodontal ligament thickness. (e) OTM group: during the application of orthodontic force, 0.9% saline was injected at 2-day intervals. Seven days after the application of orthodontic force, micro-CT showed obvious gaps between first and second molars. (g) Double-labelled immunofluorescence staining showed that, in the context of orthodontic force, the expression levels of TRAP and YAP increased in the first molar periodontal ligament; both showed high expression in the region near IL-20 expression. (h) There was no significant change in the periodontal ligament in the control group. (i) HE staining was used to measure the periodontal ligament on the compressed and traction sides of the first molar in the control and OTM groups. The periodontal ligament thickness in the OTM group was significantly thinner on the compressed side than on the traction side. Bars represent the mean ± SEM of three independent experiments (*n* = 12). ^∗^*P* < 0.05 vs. OTM group; N.S.: not significant.

The above results confirmed that IL-20 promotes osteoclast differentiation by regulating the upstream RANK/RANKL/OPG pathway and the RANKL-mediated downstream signalling pathway. “Orthodontic tooth movement” is a unique bone remodelling process within the jaw, which mainly manifests through bone formation on the tension side and bone resorption on the compression side. This mechanism has received extensive attention in the field of oral biomechanics [29]. To investigate whether IL-20 can accelerate bone resorption and bone remodelling in rats, we established a rat orthodontic tooth movement model (Figure 6(e)), with maxillary incisors as a base point, that used an orthodontic treatment spring with consistent force (50 g) along a first molar. Micro-CT of the maxilla showed that, compared with the control group, the gap between the first and second molars was increased in the OTM group; thus, the transverse and longitudinal sections of the first molars were both visible in the OTM group (Figure 6(b)). HE staining of the transverse plane of the first molars showed that the compressed side of the periodontal ligament of the molars was narrower in the OTM group than in the control group (Figures 6(d), 6(f), 6(h), and 6(i)). Based on the results of vitro experiments, we injected IL-20 into the intraperitoneal cavities of rats that had been subjected to orthodontic force. Three days before modelling, rats in the OTM + IL-20 group received IL-20 solution at a rate of 40 mg/kg body weight. Intraperitoneal injections were performed at 2-day intervals before 3 days of modelling; on the 10th day, the rats were sacrificed and micro-CT was performed (Figure 6(a)) to analyse the first molar movement distance. Compared with the OTM + vehicle group (Figure 6(c)), the OTM + IL-20 group had greater tooth movement distance over 7 days of exposure to similar force for a similar duration (Figure 6(b)); bone resorption was also greater in the OTM + IL-20 group. These findings suggested that IL-20 accelerates the formation of bone fractures and the speed of bone reconstruction. To test this hypothesis, double-labelling immunofluorescence staining of the periodontal ligament of the maxillary first molar was performed. The results indicated that the expression of IL-20 in the periodontal ligament increased after orthodontic tooth movement with IL-20 injection, compared with the control group; moreover, the expression levels of TRAP and YAP also increased in the group with IL-20 injection (Figure 6(g)). TRAP is an osteoclast-specific protein, while YAP is a protein that responds to mechanical stress. The increased expression level of IL-20 in the periodontal ligament of teeth undergoing orthodontic movement implied that IL-20 is important in alveolar bone reconstruction. The increased expression levels of TRAP and YAP in the region near IL-20 expression suggest that, while IL-20 promotes osteoclast differentiation and accelerates bone remodelling, it can also enable periodontal ligament cells to more robustly respond to orthodontic force, further accelerating alveolar bone remodelling.

## 4. Discussion

IL-20 is a powerful proinflammatory, chemotactic, and angiogenic cytokine of the IL-10 family. In chronic inflammatory diseases (e.g., psoriasis, atherosclerosis, and rheumatoid arthritis), IL-20 has been shown to exhibit robust proinflammatory, vascular regenerative, and cell chemotactic effects [30, 31]. The IL-20 family of cytokines can strengthen tissue remodelling and wound healing, maintain tissue integrity, and maintain and restore homeostasis in the context of infection and inflammation [32]. Some studies have shown that IL-20 plays important roles in osteoporosis and bone loss-related diseases; moreover, studies in ovariectomised mice have shown that anti-IL-20 antibodies can prevent bone resorption by blocking osteoclast formation and inducing osteoblast formation [17, 18]. Because of breakthroughs in the osteoclastogenesis molecular mechanism by means of coculture systems comprising BMSCs and BMMs or T cells, many cytokines and chemokines involved in bone remodelling and bone resorption have been identified; these include TNF-*α*, IL-1, IL-6, IL-10, IL-17, and IL-22 [33–40].

An IL-20 receptor (IL-20R) cytokine is reportedly expressed by immune cells. IL-20R cytokines are presumably related to the pathogenesis of chronic inflammation and autoimmune diseases. Some studies have shown that IL-20R cytokines play a suppressive role in regulating immune cells, such as innate and adaptive T cell responses [41]; thus, the functions and roles of IL-20R cytokines in autoimmunity are presumably complex. IL-20 and its family of cytokines share three receptors (IL-20RA, IL-20RB, and IL-22RA1); because of the promiscuity of the type I (IL-20RA and IL-20RB heterodimer) and type II (IL-20RAI and IL-20RB heterodimer) receptors, IL-20 and its family have some distinctive features [31]. IL-20 can signal through both type I receptor heterodimers and type II receptor heterodimers, and both receptor heterodimers share the common receptor subunit IL-20RB [42]. Therefore, IL-20RB may have an important effect on the cellular roles of IL-20.

We used siRNA knockdown to reduce the expression of IL-20 in rat BMMs and found that RANKL-induced osteoclastogenesis decreased. However, our findings demonstrated that IL-20 has a dual effect on osteoclast differentiation and function. A low concentration of IL-20 promoted both preosteoclast proliferation and osteoclastogenesis, whereas a high concentration of IL-20 inhibited BMM proliferation and osteoclastogenesis. Notably, transfection with an IL-20-overexpression plasmid did not cause inhibition of osteoclast differentiation; in contrast, it activated the RANKL-mediated osteoclastogenic downstream signalling pathway and promoted osteoclast differentiation. These findings suggest that IL-20 binds to two receptor complexes: IL-20R1/IL-20R2 and IL-22R1/IL-20R2. Both heterodimeric receptor complexes partially signal through the JAK/STAT pathway. Moreover, IL-20 binds to its receptor and enters the cell to activate STAT1. Our KEGG pathway analyses revealed that this activation of the JAK/STAT signalling pathway and STAT1 are sufficient to inhibit the osteoclast differentiation by inhibiting both c-Fos and TRAF6. We presume that the enhanced expression of IL-20 can directly upregulate on the TRAF6/NF-*κ*B/NFATc1 signal pathway to promote osteoclast differentiation.

In accordance with previous findings, we added recombinant IL-20 protein to rat BMMs that had knocked down IL-20RB and cocultured. The results suggested that the expression of NF-*κ*B was increased, although the RANKL-mediated TRAF6/NFATc1 signalling pathway did not significantly change. Thus, in the absence of IL-20RB, IL-20 does not influence osteoclast differentiation. These data indicate that IL-20RB has an important effect on the cellular functions of IL-20. Our findings indicate that IL-20RB offers a potential therapeutic target for patients with bone loss disease and osteoporosis, which may effectively inhibit bone loss.

Osteoblast–osteoclast communication, regulated by various molecules, cytokines, and signalling pathways, is important for bone homeostasis. The OPG/RANK/RANKL axis is an important signalling pathway in this communication, and IL-20 is an important regulator of the balance between osteoblastogenesis and osteoclastogenesis [37, 38]. Studies have shown that bone-associated immune mediators target IL-20 in MC3T3-E1 cells (mouse osteoblasts) and mature osteoclasts; moreover, IL-20 acts as an important regulator of osteoblasts and osteoclasts by activating OPG/RANK/RANKL, which are essential components in the osteoclast signalling pathway [43, 44]. In this study, we confirmed that IL-20 can induce BMSCs to regulate the expression of OPG and RANKL and then affect osteoclastogenesis through the OPG/RANKL/RANK axis. Subsequently, we found that the RANKL/TRAF6/NF-*κ*B and JNK/p38/MAPK signalling pathways were activated during osteoclast differentiation in BMMs, following culture with CM from IL-20-overexpressing BMSCs. However, the RANKL/TRAF6/NF-*κ*B and JNK/p38/MAPK signalling pathways were not activated during osteoclast differentiation in BMMs, following culture with CM from IL-20 and IL-20RB knockdown BMSCs. Overall, our findings indicated that RANKL and OPG in BMSCs were endogenously induced by IL-20, which supports the notion of regulatory feedback during osteoclast differentiation through the OPG/RANKL/RANK axis.

“Orthodontic tooth movement” is the application of appropriate “biological force” to the teeth, alveolar bone, and jaw to cause physiological movement, thereby correcting malocclusion (occlusion) deformities. The imbalance between osteoblasts and osteoclasts is the biological basis of tooth movement and alveolar bone reconstruction [29]. Through in vitro experiments, we clarified the effects of IL-20 and its key receptor, IL-20RB, on osteoclast differentiation and functions. Additionally, we found that IL-20 stimulates BMSCs to participate in feedback regulation of osteoclast formation through the OPG/RANKL/RANK axis. Moreover, IL-20 can change the local immune environment and affect osteoclast differentiation. Our in vitro findings implied that IL-20 can affect orthodontic tooth movement. Using a rat model of tooth movement, we injected IL-20 solution into the abdominal cavity. Notably, rats that had been injected with IL-20 solution exhibited significant enhancements of tooth movement speed and distance. These findings indicate that IL-20 accelerates the speed of osteoclast differentiation and alveolar bone reconstruction. Through double-labelling immunofluorescence staining of the periodontal ligament during orthodontic tooth movement, we found that IL-20 in the periodontal ligament is closely associated with the osteoclast-specific protein, TRAP, and the mechanical stress response protein, YAP. Thus, IL-20 can promote osteoclast differentiation and accelerate the speed of orthodontic tooth movement; it can also enable the periodontal ligament to better respond to the mechanical force of the load and accelerate the reconstruction of alveolar bone.

In conclusion, we found that IL-20 can differentially regulate osteoclast formation and osteoclast-mediated bone resorption capacity. IL-20 acts on the upstream differential regulation of primary cell osteoclastogenesis by regulating the OPG/RANKL/RANK axis. In addition, we demonstrated that IL-20 may activate the OPG/RANKL/RANK axis, and we revealed a possible molecular mechanism involving the RANKL/NF-*κ*B/NFATc1 pathway. Using IL-20 at a concentration of 20 ng/mL, combined with siRNA transfection to reduce the expression of IL-20RB, a key receptor of IL-20, we achieved partial inhibition of the RANKL-mediated downstream signalling pathway regulated by IL-20. We also used high-throughput transcriptome sequencing to confirm that enhanced expression of IL-20 can substantially influence the cellular immune response, stimulus feedback, and cellular response to stress; these effects are in addition to its role in the osteoclast differentiation pathway. Our in vivo analyses confirmed that IL-20 could promote osteoclast differentiation, thereby accelerating the speed of orthodontic tooth movement. In addition, IL-20 promoted the expression of mechanical force response proteins, which may enable the periodontal ligament to more effectively adapt to the mechanical force load and may improve the speed of bone remodelling. Our findings imply that targeting IL-20 may be a promising therapeutic approach for diseases with bone loss; they also support a new perspective regarding the investigation of orthodontic tooth movement.

## Figures and Tables

**Table 1:** Primer sequences used for real-time PCR.

Gene	Forward primer sequence (5′-3′)	Reverse primer sequence (5′-3′)
GAPDH	GGCACAGTCAAGGCTGAGAATG	ATGGTGGTGAAGACGCCAGTA
IL-20	ACTGCAAACCTACAGGCGATACAA	AGAACCTCACTAGATGGCGGAGA
IL-20RB	AGCACTTGATGGGTTAACAGCC	GAAAACAGAGACACAGCCCTCC
RANK	CAGGACAGGGCTGATGCAA	TGACTGACGTACACCACGATGA
RANKL	CTCATGCAGGAGAATCAAAC	TTCCATCATAGCTGGAACTC
OPG	GACCAAAGTGAATGCCGAGAG	CGCTGCTTTCACAGAGGTCAA

**Table 2:** siRNA IL-20.

Target gene name	Target gene sequence
si-r-Il20_001	CAACCAAATTCTCAGTCAT
si-r-Il20_002	GGATGGAGGAGATGTTATA
si-r-Il20_003	CTTCTCTGGACTCCTTTAA

**Table 3:** siRNA IL-20RB.

Target gene name	Target gene sequence
si-r-Il20rb_001	TCTCTGTACGGTCAACCAA
si-r-Il20rb_002	ATTCCGGTGCACCTAGAAA
si-r-Il20rb_003	CCTGACACCTTGAAAGTAA

**Table 4:** Overexpression plasmid.

Target sequence name	r-Il20 (NM_001143881.1 complete CDS)-WT
Clone length	518 bp (NM_001143881.1 complete CDS)
5′ restriction site	EcoRI
3′ restriction site	XhoI
Vector name	pEXP-RB-Mam-EGFP
Frame size	4.8 kb
Host bacteria	*E. coli* DH5*α*
Antibiotic resistance	Ampicillin
Copy number	High copy

## Data Availability

The data that support the findings of this study are available from the corresponding author upon reasonable request.

## Abbreviations

IL: Interleukin
siRNA: Small interfering RNA
mRNA: Messenger ribonucleic acid
GO: Gene Ontology
KEGG: Kyoto Encyclopedia of Genes and Genomes
OTM: Orthodontic tooth movement
qRT-PCR: Real-time quantitative polymerase chain reaction
FBS: Fetal bovine serum
cDNA: Complementary DNA
PBS: Phosphate buffer saline
DMEM: Dulbecco's modified eagle medium
OMEM: Opti modified eagle medium
SD: Standard deviation
M-CSF: Macrophage colony-stimulating factor
RANK: Receptor activator of NF-кB
RANKL: Receptor activator of NF-кB ligand
CCK-8: Cell counting kit-8
OverEx: Overexpression
OPG: Osteoclastogenesis inhibitory factor
TRAP: Tartrate resistant acid phosphatase
CTSK: Cathepsin K
MMP-9: Matrix metalloprotein-9
RBC: Red blood cell
BMSC: Bone marrow mesenchymal stem cell
BMM: Marrow-derived macrophage
RIPA: Radio immunoprecipitation assay
SDS-PAGE: Sodium dodecyl sulfate-polyacrylamide gel electrophoresis
BSA: Bovine albumin
IL-20RA: Interleukin 20 receptor subunit alpha
IL-20RB: Interleukin 20 receptor subunit beta
IL-22RA1: Interleukin 22 receptor subunit alpha 1
GRB2: Growth factor receptor-bound protein 2
ERK: Extracellular regulated MAP kinase
JNK: c-Jun N-terminal kinase
TRAF6: Tumor necrosis factor receptor-associated factor 6
IKK: Inhibitor of kappa B kinase beta
NFATc1: Nuclear factor of activated T cells 1
p-38: p38 kinase
HIF-*α*: Hypoxia-inducible factor 1 subunit alpha a
CXCR4: C-X-C motif chemokine receptor 4
CCR7: C-C motif chemokine receptor 7
VEGF-R2: Vascular endothelial growth factor receptor 2.

## References

[1] Hassan A. H., Al-Saeed S. H., Al-Maghlouth B. A., Bahammam M. A., Linjawi A. I., El-Bialy T. H. (2015). Corticotomy-assisted orthodontic treatment. *Saudi Medical Journal*.

[2] Taddei S. R., Andrade I., Queiroz-Junior C. M. (2012). Role of CCR2 in orthodontic tooth movement. *American Journal of Orthodontics and Dentofacial Orthopedics*.

[3] Suchacki K. J., Roberts F., Lovdel A. (2017). Skeletal energy homeostasis: a paradigm of endocrine discovery. *The Journal of Endocrinology*.

[4] Crockett J. C., Rogers M. J., Coxon F. P., Hocking L. J., Helfrich M. H. (2011). Bone remodelling at a glance. *Journal of Cell Science*.

[5] Matsuo K., Irie N. (2008). Osteoclast-osteoblast communication. *Archives of Biochemistry and Biophysics*.

[6] Ushach I., Zlotnik A. (2016). Biological role of granulocyte macrophage colony-stimulating factor (GM-CSF) and macrophage colony-stimulating factor (M-CSF) on cells of the myeloid lineage. *Journal of Leukocyte Biology*.

[7] Stanley E. R., Cifone M., Heard P. M., Defendi V. (1976). Factors regulating macrophage production and growth: identity of colony-stimulating factor and macrophage growth factor. *Journal of Experimental Medicine*.

[8] Simonet W. S., Lacey D. L., Dunstan C. R. (1997). Osteoprotegerin: a novel secreted protein involved in the regulation of bone density. *Cell*.

[9] Tsuda E., Goto M., Mochizuki S. (1997). Isolation of a novel cytokine from human fibroblasts that specifically inhibits osteoclastogenesis. *Biochemical and Biophysical Research Communications*.

[10] Yasuda H., Shima N., Nakagawa N. (1998). Identity of osteoclastogenesis inhibitory factor (OCIF) and osteoprotegerin (OPG): a mechanism by which OPG/OCIF inhibits osteoclastogenesis in vitro. *Endocrinology*.

[11] Tsukasaki M., Takayanagi H. (2019). Osteoimmunology: evolving concepts in bone-immune interactions in health and disease. *Nature Reviews. Immunology*.

[12] Kim K. W., Kim H. R., Park J. Y. (2012). Interleukin-22 promotes osteoclastogenesis in rheumatoid arthritis through induction of RANKL in human synovial fibroblasts. *Arthritis and Rheumatism*.

[13] Schulze J., Bickert T., Beil F. T. (2011). Interleukin-33 is expressed in differentiated osteoblasts and blocks osteoclast formation from bone marrow precursor cells. *Journal of Bone and Mineral Research*.

[14] Xue Y., Liang Z., Fu X., Wang T., Xie Q., Ke D. (2019). IL-17A modulates osteoclast precursors' apoptosis through autophagy-TRAF3 signaling during osteoclastogenesis. *Biochemical and Biophysical Research Communications*.

[15] Yokota K., Sato K., Miyazaki T. (2014). Combination of tumor necrosis factor *α* and interleukin-6 induces mouse osteoclast-like cells with bone resorption activity both in vitro and in vivo. *Arthritis & Rhematology*.

[16] Paradowska-Gorycka A., Grzybowska-Kowalczyk A., Wojtecka-Lukasik E., Maslinski S. (2010). IL-23 in the Pathogenesis of Rheumatoid Arthritis. *Scandinavian Journal of Immunology*.

[17] Hsu Y. H., Chen W. Y., Chan C. H., Wu C. H., Sun Z. J., Chang M. S. (2011). Anti–IL-20 monoclonal antibody inhibits the differentiation of osteoclasts and protects against osteoporotic bone loss. *Journal of Experimental Medicine*.

[18] Hsu Y. H., Chiu Y. S., Chen W. Y. (2016). Anti-IL-20 monoclonal antibody promotes bone fracture healing through regulating IL-20-mediated osteoblastogenesis. *Scientific Reports*.

[19] Dandajena T. C., Ihnat M. A., Disch B., Thorpe J., Currier G. F. (2012). Hypoxia triggers a HIF-mediated differentiation of peripheral blood mononuclear cells into osteoclasts. *Orthodontics & Craniofacial Research*.

[20] Huang H., Williams R. C., Kyrkanides S. (2014). Accelerated orthodontic tooth movement: molecular mechanisms. *American Journal of Orthodontics and Dentofacial Orthopedics*.

[21] Park H. J., Baek K. H., Lee H. L. (2011). Hypoxia inducible factor-1*α* directly induces the expression of receptor activator of nuclear factor-*κ*B ligand in periodontal ligament fibroblasts. *Molecules and Cells*.

[22] Maridas D. E., Rendina-Ruedy E., Le P. T., Rosen C. J. (2018). Isolation, Culture, and Differentiation of Bone Marrow Stromal Cells and Osteoclast Progenitors from Mice. *Journal of Visualized Experiments*.

[23] Takahashi N., Akatsu T., Udagawa N. (1988). Osteoblastic cells are involved in osteoclast formation. *Endocrinology*.

[24] Yoshida H., Hayashi S., Kunisada T. (1990). The murine mutation osteopetrosis is in the coding region of the macrophage colony stimulating factor gene. *Nature*.

[25] Yasuda H., Shima N., Nakagawa N. (1998). Osteoclast differentiation factor is a ligand for osteoprotegerin/osteoclastogenesis-inhibitory factor and is identical to TRANCE/RANKL. *Proceedings of the National Academy of Sciences*.

[26] Suda T., Takahashi N., Udagawa N., Jimi E., Gillespie M. T., Martin T. J. (1999). Modulation of osteoclast differentiation and function by the new members of the tumor necrosis factor receptor and ligand families. *Endocrine Reviews*.

[27] Kong Y. Y., Yoshida H., Sarosi I. (1999). OPGL is a key regulator of osteoclastogenesis, lymphocyte development and lymph-node organogenesis. *Nature*.

[28] Lacey D. L., Boyle W. J., Simonet W. S. (2012). Bench to bedside: elucidation of the OPG–RANK–RANKL pathway and the development of denosumab. *Nature Reviews Drug Discovery*.

[29] Li Y., Jacox L. A., Little S. H., Ko C. C. (2018). Orthodontic tooth movement: the biology and clinical implications. *The Kaohsiung Journal of Medical Sciences*.

[30] Autieri M. V. (2018). IL-19 and Other IL-20 Family Member Cytokines in Vascular Inflammatory Diseases. *Frontiers in Immunology*.

[31] Wegenka U. M. (2010). IL-20: biological functions mediated through two types of receptor complexes. *Cytokine & Growth Factor Reviews*.

[32] Ouyang W., Rutz S., Crellin N. K., Valdez P. A., Hymowitz S. G. (2011). Regulation and functions of the IL-10 family of cytokines in inflammation and disease. *Annual Review of Immunology*.

[33] Medhat D., Rodríguez C. I., Infante A. (2019). Immunomodulatory Effects of MSCs in Bone Healing. *International Journal of Molecular Sciences*.

[34] Lieder R., Sigurjonsson O. E. (2014). The effect of recombinant human interleukin-6 on osteogenic differentiation and YKL-40 expression in human, bone marrow-derived mesenchymal stem cells. *Biores Open Access*.

[35] Ma L., Aijima R., Hoshino Y. (2015). Transplantation of mesenchymal stem cells ameliorates secondary osteoporosis through interleukin-17-impaired functions of recipient bone marrow mesenchymal stem cells in MRL/lpr mice. *Stem Cell Research & Therapy*.

[36] El-Zayadi A. A., Jones E. A., Churchman S. M. (2016). Interleukin-22 drives the proliferation, migration and osteogenic differentiation of mesenchymal stem cells: a novel cytokine that could contribute to new bone formation in spondyloarthropathies. *Rheumatology*.

[37] Liu H., Xu G. W., Wang Y. F. (2015). Composite scaffolds of nano-hydroxyapatite and silk fibroin enhance mesenchymal stem cell-based bone regeneration via the interleukin 1 alpha autocrine/paracrine signaling loop. *Biomaterials*.

[38] Chen Z., Su L., Xu Q. (2015). IL-1R/TLR2 through MyD88 divergently modulates osteoclastogenesis through regulation of nuclear factor of activated T cells c1 (NFATc1) and B lymphocyte-induced maturation protein-1 (Blimp1). *Journal of Biological Chemistry*.

[39] Tanaka K., Yamagata K., Kubo S. (2019). Glycolaldehyde-modified advanced glycation end-products inhibit differentiation of human monocytes into osteoclasts via upregulation of IL-10. *Bone*.

[40] Udagawa N., Takahashi N., Jimi E. (1999). Osteoblasts/stromal cells stimulate osteoclast activation through expression of osteoclast differentiation factor/RANKL but not macrophage colony- stimulating factor. *Bone*.

[41] Chen J., Caspi R. R., Chong W. P. (2018). IL-20 receptor cytokines in autoimmune diseases. *Journal of Leukocyte Biology*.

[42] Logsdon N. J., Deshpande A., Harris B. D., Rajashankar K. R., Walter M. R. (2012). Structural basis for receptor sharing and activation by interleukin-20 receptor-2 (IL-20R2) binding cytokines. *Proceedings of the National Academy of Sciences*.

[43] Zhang W., Magadi S., Li Z., Smith C. W., Burns A. R. (2017). IL-20 promotes epithelial healing of the injured mouse cornea. *Experimental Eye Research*.

[44] Ikebuchi Y., Aoki S., Honma M. (2018). Coupling of bone resorption and formation by RANKL reverse signalling. *Nature*.

